# Clean Label in Bread

**DOI:** 10.3390/foods10092054

**Published:** 2021-08-31

**Authors:** Maite Cristina Alava Vargas, Senay Simsek

**Affiliations:** 1 Cereal Science Graduate Program, Department of Plant Sciences, North Dakota State University, Fargo, ND 58108-6050, USA; maite.alavavargas@ndus.edu; 2 Department of Food Science, 745 Agricultural Mall Drive, Purdue University, West Lafayette, IN 47907, USA

**Keywords:** bread, clean label, preservatives, dough strengtheners

## Abstract

Bread is considered a staple food worldwide, and therefore there is much interest in research around the topic. The bread industry is usually looking for ways to improve its formulations. Therefore, other ingredients such as dough conditioners, crumb softeners, emulsifiers, and surfactants can be added to enhance bread quality. These ingredients perform functions such as helping standardize processes in the industry, reducing dough-mixing time, increasing water absorption, improving bread quality, and extending its shelf life. Consumers are concerned about the effect of these ingredients on their health, and this has increased the popularity of clean-label bread formulations. A clean label generally indicates that a product is free of chemical additives, has an ingredient list that is easy to understand, has undergone natural or limited processing, and/or is organic and free of additives or preservatives. However, there is no scientific definition of the term “clean label.” Researchers have focused on these clean-label initiatives to replace dough strengtheners and preservatives in bread formulations and give consumers what they perceive as a healthier product.

## 1. Introduction and Target of This Review

Bread is a centuries-old food and considered an essential dietary staple food worldwide. In addition, what is considered “bread” and the ingredients used change depending on the region. Bread is essential in the human diet and a good source of carbohydrates, dietary fiber, protein, minerals, and vitamins [1]. Bread making has been changing over time, and there are differences in formulations, depending on the final product. Bread has four necessary ingredients: flour, yeast, water, and salt. The bread industry is usually looking for ways to improve its formulations [2,3]. In the early twentieth century, the bread industry continued to develop, and the market changed with a more standardized process. Products and processes such as bleached white flour, flour enrichment, and chemicals were added to maintain consistency and improve quality [4]. Bread formulations also have optional ingredients such as sugar (sweeteners in general), milk solids, fats, and conditioners [5]. These ingredients affect the quality and taste of the bread, but other ingredients can improve quality, enhance dough handling, and extend the shelf life [2]. Other ingredients such as oxidizing agents, reducing agents, buffers, enzymes, gluten proteins, and dough improvers/conditioners can improve dough rheology and extend the shelf life [2].

It is vital in bread making to establish the quality of flour and the final goal, considering its quality or industrial requirements to pick the right ingredients. The performance of bread made with wheat flour is directly affected by gluten proteins. Wheat flour is mainly composed of proteins, carbohydrates, and lipids [6]. The gluten proteins interact with other components of the wheat flour. When mixed with the right amount of water, the gluten matrix develops, which allows the dough to hold gas during fermentation [5,7]. The gluten proteins play a crucial role in baking by affecting the water absorption capacity, cohesivity, viscosity, and elasticity of the dough [8]. Nevertheless, when the flour’s quality varies or is lower, dough improvers help standardize the final product and preserve quality. In addition, preservatives are sometimes added to the bread formulation to extend the shelf life. Fungal spoilage in bread is commonly known and has a substantial economic impact caused by the bread becoming unsuitable for human consumption due to food safety concerns [9].

Dough improvers and conditioners have been part of formulations in the bread industry for decades. Bread improvers help overcome deficiencies in the flour’s quality, improving loaf volume, crumb structure, shelf life, flavor, and color. The conditioners can be chemical redox agents and enzymes that may or may not be endogenous to the wheat flour, influencing the gluten network [10]. The bread industry and formulations have changed in the past years due to more consumers being concerned about the food ingredients. Some bread improvers are perceived as unknown and harmful chemicals, and several may have controversial health issues. People want to know what the ingredients are and understand or feel familiar with them due to health concerns. A clean-label product can be referred to as food or ingredients that are more natural, organic, or not chemical-sounding and free of additives/preservatives [11,12,13]. The bread industry and bakeries are going for clean-label products, adding only natural ingredients and additive-/preservative-free formulations but keeping the bread quality high [14,15]. Thus, more research on natural ingredients can give the same attributes as traditional preservatives/additives to bread.

## 2. Bakery Improvers

A good-quality gluten network is highly essential in bread making, directly affecting the ability to retain the yeast’s carbon dioxide and enhancing the dough’s properties. The gluten proteins’ quality can vary because of genetic, environmental, and post-harvest conditions. To maintain the same quality in bread making, the addition of additives/preservatives, dough conditioners, or strengtheners helps overcome the deficiencies in wheat quality [10]. These dough strengtheners’ and preservatives’ primary functions are to increase machine, and mixing, and handling tolerance. Some of the process improvements lead to less time for the dough to develop its gluten matrix, maximize water absorption; minimize the use of shortening; improve bread qualities such as loaf volume, texture, and crust tenderness; retard the rate of staling; and enhance the shelf life and self-quality. Many chemical additives can be used in formulations and with different objectives, as shown in Table 1.

Different strengtheners such as potassium bromate (PB), iodate, chlorine dioxide, chlorine, azodicarbonamide (ADA), ascorbic acid (AA), diacetyl tartaric acid esters of mono- and diglycerides (DATEM), sodium stearoyl lactylate (SSL), and peroxides, alter gluten proteins [2,10,17]. Besides the effect they have on the overall dough rheology, to extend the shelf life, other chemical additives are used, too. Emulsifiers are a type of dough strengthener that improve the dough’s machinability, reduce resting time, and improve bread qualities such as volume, crust color, crumb whiteness, aroma, and flavor [18,19]. Another benefit of emulsifiers is that they can improve the products’ shelf life. Table 1 shows some of the examples of different emulsifiers and the user level. Chemical preservatives, like organic acids, are commonly used to prevent undesired microorganisms’ growth and extend the bakery products’ shelf life.

### 2.1. Diacetyl Tartaric Acid Esters of Mono- and Diacylglycerides (DATEM)

DATEM are esters of polyvalent alcohols that consist of glycerol derivates esterified with edible fatty acids and mono- and diacetyl tartaric acids. There could be an inter- and intra-exchange of acylic groups, resulting in small amounts of free glycerol, free fatty acids, and free tartaric and acetic acids [20]. DATEM’s advantages are reducing baking fat, improving chewing and taste properties, and increasing resistance during processing [18,20]. Some industries have also changed from DATEM to lipase enzymes, but more information and studies are still needed to evaluate its use in bread characteristics [19].

### 2.2. Azodicarbonamide (ADA)

Azodicarbonamide (ADA) is an oxidizing agent used in bread formulation as a dough improver because of its fast oxidation at a dosage from 2 to 45 ppm [10]. This dough improver can enhance the dough and baking qualities by oxidizing sulfhydryl groups that can tolerate high water absorption and shortened mixing times. Bread made with ADA can have a higher loaf volume and better texture, and high dosage can influence the flour color. The overdosage of this compound can result in the opposite effect by decreasing the bread quality. The dosage will vary depending on the grade of flour [10].

### 2.3. Ascorbic Acid (AA)

L-ascorbic acid is an oxidant used as a dough improver [10]. It is considered an intermediate oxidizer and promotes dough development at a high mixing speed. In addition, it improves the dough strength, reduces dough stickiness, and improves gas retention, resulting in higher loaf volumes and good crumb texture [10,13]. AA is also commonly known as vitamin C and is frequently used to replace bromate in countries where it is banned, even though it is less effective. This dough improver effect depends on the quality of the flour, storage time, bread-making procedure, type of bread, and dosage from 30 to 120 ppm. For consumers, it also gives an effect of freshness in the bread by better recovery of the bread shape after compression [10,13].

### 2.4. Potassium Bromate (PB)

Potassium bromate is an oxidant, usually used at a level of 10 ppm on a flour basis [13]. It influences the loaf volume and improves dough expansion, crumb structure, and texture. PB has an inhibitory effect on the proteolytic enzymes in wheat flour and is considered a slow-acting oxidant. The bromate oxidizes free sulfhydryl groups, generating disulfide (SS) compounds and bromide [10,13]. It is also more active at later stages of fermentation and baking, improving dough expansion. Bromate enhancement of the rheological properties will depend on the flour characteristics and the amount added [10,13].

### 2.5. Propionic and Sorbic Acids

Propionic and sorbic acids are commonly used, but other acids, such as benzoic acid, are examined for bakery products with restrictions in their use. In prepacked unsliced bread, 0.2% (*w*/*w*) of sorbate and 0.3% of propionate can be used, whereas in unpacked bread, the addition of neither sorbate nor propionate is allowed [21]. Propionic acid and others have an antimicrobial action and are used to preserve baked goods [22]. High concentrations are not recommended for sorbate or propionate as additives because they alter the product [21]. Propionic acid is highly volatile in steam, and sorbic acid is not frequently soluble in water. In addition, using dry sorbic acid is less recommended because it irritates the skin and mucous membrane. Sorbic acid and calcium sorbate are also used in the food industry as active substances in fungistatic film packaging [23].

## 3. Health Effects

Consumers are looking for natural ingredients and no chemical additives due to health concerns. Most of the additives used nowadays do not have a health risk, but still, some consumers prefer the use of familiar names in the list of ingredients. Some of these additives/preservatives show health risks if used in specific ways or in excess. The use of ADA can impose a health risk during bread making if transformed into semicarbazide (SEM), which can be exceptionally high in the crust and have mutagenic and carcinogenic effects [10]. During baking, ADA is converted to biurea, which is partly converted to SEM. This dough improver is prohibited in the European Union, but other countries such as Brazil, the United States, and Canada still permit it. PB is also an additive considered to be carcinogenic. In rats, when given orally, it has been proven to induce renal cell tumors, mesotheliomas of the peritoneum, and follicular cell tumors of the thyroid [24]. After baking, there can still be residual bromate in the bread crust with a usage of 9 ppm. The usage of PB is banned in some countries, while in others it is still legal, but its usage has reduced significantly [10]. AA is considered an essential dietary nutrient and acts like an antioxidant, but it can also cause various chronic diseases if misused [25]. However, in studies with a high dosage of vitamin C, being 2 g per day, it is not likely to pose a risk or adverse effects in most individuals. DATEM, another dough strengthener used in bread formulations, may have toxicity in rats, causing heart fibrosis and adrenal overgrowth [26].

## 4. Consumer Perception

Shoppers currently tend to purchase food products that they feel good about and believe are healthy, not only based on nutrition, but also based on being familiar with the ingredients [13,27,28]. In Europe, 78% of shoppers consider the ingredients a vital factor when deciding on a product [13]. In addition, some consumers can react adversely to products containing ingredients with unknown names, scientific names, or names that confuse them. Shoppers have become more skeptical, and if certain ingredients are not recognized, certain products can be seen as too processed and sometimes even dangerous [29]. Younger-generation shoppers spend more time going through a product before deciding to buy it and are more likely to buy new, trendy, and attractive products [28]. Other products that are shown to be organic or natural, environmentally friendly, socially sustainable, and innovative are also captivating buyers’ attention. Around 56% of consumers look for clean-label products because they are thought to be more natural and healthier [15]. Different ingredients such as ascorbic acid, whey protein, and starch are more accepted in the ingredient list than guar gum, xantham gum, caseinate, and others [13]. Consumers still seek healthier products that maintain freshness, quality, taste, texture, and consistency at an affordable price. The challenge for more industries is trying to look for clean-label reformulations that match the efficiency, low cost, and ease of reading of ingredients that consumers look for without lowering their quality. Product consistency is what most buyers look for in a product, and bakers seek formulations with better dough handling and shelf-life stability [27].

## 5. Clean-Label

**Figure 1:**
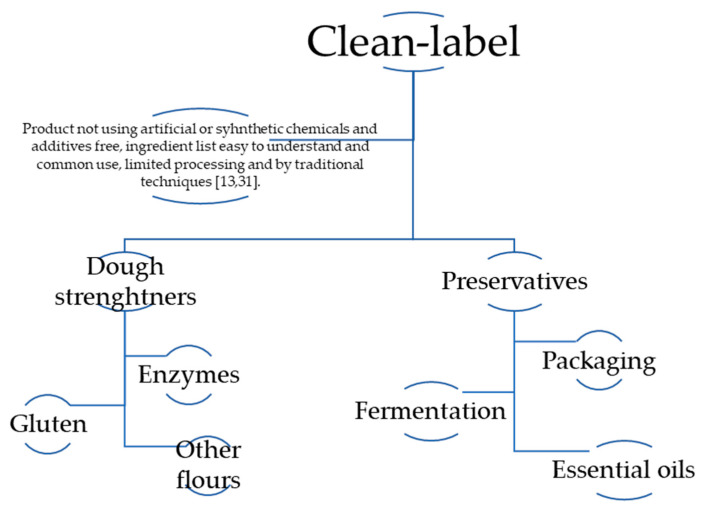
An example of the clean-label definition and possible solutions in clean-label bread formulations [12,13,31].

Clean-label trends have been continuously increasing in the past few years, and regulations depend on the region [13]. Consumers are more concerned about having a healthier life, and in the United States, sales of natural bread and baking goods saw an overall growth of 11.3% in the last year up to March 2020 [30]. In Europe, consumers are concerned about the ingredient list of a product, which has made the clean label a market success [14]. There is no scientific definition of what is considered clean-label, but there is still a proposed definition, as shown in Figure 1.

A clean-label product must be natural, organic, and/or free from additives/preservatives [13]. At the same time, other markets just refer to it as ingredients that are understandable, that are easy to recognize without being a food scientist, and that consumers believe are healthful and not harmful [14,27]. Most of them have similarities in how a clean label is defined, but it can be seen in two different ways. Institutions such as Ingredion define a clean-label product as having natural, organic, or wholesome constituents; the names of the ingredients are not chemical-sounding and are familiar to the consumer; and the constituents are considered ingredients (food) rather than chemicals [13,14]. Other institutions also add fewer total ingredients—ingredients that positively contribute to health and the absence of constituents that contribute to an unhealthy lifestyle, such as fat [11].

In a broad sense, a clean-label product will have written or visual claims of being clean-label, have certification logos, have a simple front of package labels, belong to natural and organic categories, and be free from preservatives/additives [32]. A clean-label product, in the strict sense, will have a detailed ingredient list and a nutrition facts panel; a short, simple ingredient list with familiar ingredients; and no artificial or chemical-sounding ingredients [32]. Even though consumers are looking for what they think are healthier products, such as those with clean-label formulations, the products must still maintain freshness, good quality, taste, texture, and consistency [27]. In recent years, different industries have focused their research on ingredients that fit these characteristics and replace chemical additives/preservatives. Natural ingredients as dough strengtheners and preservatives have become a significant alternative for clean-label formulations.

### 5.1. Clean-Label Dough Strengtheners

In bread formulations, dough strengtheners improve the dough-handling process and hydration, increase the volume and/or crumb texture, reduce staling, and improve nutritional qualities [17]. There are different chemical ingredients used for this purpose in the industry. Now more research is conducted to find other improving agents with more natural-sounding ingredients in clean-label formulations. Research has been conducted on the use of wheat flours and other flours and enzymes as alternatives [16,17,29].

#### 5.1.1. Wheat Gluten

Vital gluten is considered a highly valued functional protein and is the insoluble protein portion of wheat flour. In bakery formulations, vital wheat gluten is essential due to its features such as increasing functional protein content, water absorption, dough tolerance, and viscoelasticity, improving the volume and end product quality [33,34]. The gluten protein network of wheat flour is divided into monomeric gliadins and polymeric glutenins. Most gliadins contain intramolecular disulfide (SS) bonds, and glutenin subunits also form intermolecular SS bonds that stabilize glutenin polymers. In the dough, the hydrated gliadins are responsible for dough extensibility and viscosity and are less cohesive than glutenins [8].

In contrast, hydrated glutenin is more responsible for dough strength and elasticity due to its cohesiveness and elasticity [8]. A proper mixture of gliadins and glutenins is essential for the dough’s viscoelastic properties and quality for a starch–gluten matrix that can hold gas cells for better crumb and large loaf volumes of bread [35]. High-quality, hard red spring wheat flour and vital gluten can substitute dough strengtheners as an option for clean-label formulations [16,27,34]. The dough rheology and bread quality correlate with the molecular weight distribution of protein extracted from hard red spring wheat. Low-molecular-weight glutenin subunits (LMW-GS) strongly influence the bread-making quality [16,36]. Different researchers have shown that vital gluten is essential in bakery applications used as a binding agent, contributing to dough strength, gas retention, and rise, while improving texture and flavor [34].

#### 5.1.2. Other Strengtheners

**Figure 2:**
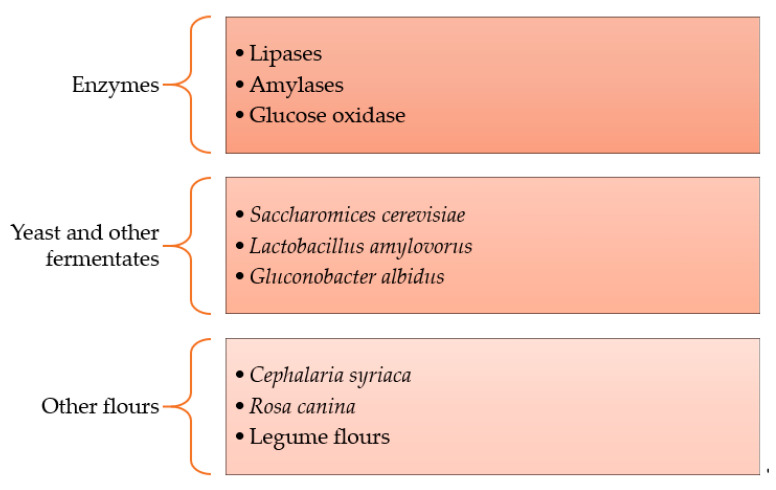
Summary of other strengthener options for clean-label formulations.

In bread formulations, dough strengtheners have become necessary for the handling process and to obtain a standardized product all year long. Some companies have started using advanced enzyme oxidant systems and other ingredients to meet clean-label standards (Figure 2) [29]. Enzymes are seen as more natural and fit clean-label standards. Hydrocolloids and emulsifiers can be replaced by enzymes and used as an alternative to chemical-improving agents [17]. In flours, some enzymes occur naturally, and in others, they can be added to increase dough handling and hydration, improve volume and/or crumb structure, reduce staling, or improve nutritional qualities [17]. Lipase enzymes have recently been used in the industry as a replacement for DATEM [18]. Some of the reasons are difficulties in DATEM transportation and cost and more bakers looking for alternatives for emulsifiers in clean-label formulations [18,27].

Amylases affect the gluten–starch matrix and, in the end, have been proven to affect the crumb structure, increase the level of fermentable sugars, and increasing the loaf volume, and heat-stable amylases help reduce staling in bread [37,38]. Some researchers have investigated adding malt flour to whole-wheat bread instead of pure α-amylase due to the abundant amount of this compound [17]. The effectiveness of malt flour depends on the flour quality. Malt flour can increase water absorption and extensibility, but it can also weaken the dough and decrease crumb quality. In [39], malt flour marginally increased the loaf volume, but it was still a significant increase (*p* < 0.05) compared to the control.

Another enzyme that is used as an oxidizing agent in improving bread quality is glucose oxidase. This enzyme catalyzes the oxidation of glucose into gluconic acid and hydrogen peroxide [17]. Glucose oxidase has been shown to affect dough extensibility in whole-wheat bread and decrease the energy for handling during bread production to a similar level as white dough. In addition, glucose oxidase has been shown to increase loaf volume more in whole-wheat bread than in white bread without additional improvers or added gluten [17].

Besides malt flour, there have been studies on adding other types of flour or using different yeast species and levels as strengtheners in bread formulations [40]. A higher level of yeast can affect the specific loaf volume in some formulations. *Lactobacillus* species are used in clean-label breads to inhibit bacterial growth, and strains such as *Lactobacillus amylovorus* as a culture starter in wheat dough fermentation show good rheological properties in bread. In buckwheat sourdough bread, other fermenters, such as *Gluconobacter albidus* (TMW 2.1191) and *Kozakia baliensis* (NBRC 16680), also improved the bread sensory properties, with a high specific volume and softer crumb [41].

Some researchers have found a use for defatted *Cephalaria syriaca* flour as a dough strengthener. *Cephalaria syriaca* is a plant that is prominent in Turkey in wheat fields and whose planting is usually encouraged. This flour has been shown to increase weak flour’s strength, positively affecting extensograph characteristics. Another flour that is used as a strengthener is rosehip (*Rosa canina*) pip flour. This plant is well known to have elevated vitamin C content, which has proven to improve dough rheological properties instead of synthetic ascorbic acid. In addition, rosehip can be a source of other vitamins and minerals, carotenoids, tocopherol, organic acids, amino acids, and essential oils [33,39]. Legume flour has also been a focus of research due to its nutritional quality. Besides its contribution of vitamins and minerals, its enzyme activity has been shown to increase the specific volume of whole-wheat bread due to its lipoxygenase activity [16]. Other researchers have also found that inactive yeast can be used as a dough relaxer and is able to replace additives such as L-cysteine and sodium metabisulfite [14].

### 5.2. Clean-Label Preservatives

Preservatives are vital in the bread industry to maintain the freshness and quality of bread for extended periods. Mold growth is one of the most critical challenges in bread’s shelf life, *Penicillium* spp. and *Aspergillus* spp. being the most dominant species [42]. In bakery products, shelf life is expected to be between 3 and 4 days when they are unpreserved, and spoilage after this period is commonly due to fungi [43]. Food preservation helps reduce fungal spoilage and loss of quality; however, more consumers have a negative perspective toward food preservatives. More people look for natural antimicrobial preservatives instead of chemical-sounding ones, such as propionic and sorbic acids and salts [44]. Some researchers have tested ingredients such as essential oils and fermentates to see whether they can have the same antimicrobial effect in bakery products as chemical preservatives. Other ways to preserve bread are using lactic acid ingredients to control the bread’s pH, being highly effective against pathogens [27].

#### 5.2.1. Fermentation

Innovation in fermentation technology is one response to bakers’ challenges of finding low-cost and natural ingredients in clean-label bread. Fermentation is a natural process in baking, and it contributes to flavor development and texture-generating organic acids. One of the baker’s effective ways to control pH in bread products is using lactic acid solutions, which also helps as an effective antimicrobial barrier [27]. In studies aiming to find new antispoilage methods, sourdough fermentation has shown promising results. Using different *Lactobacillus* spp. as starter cultures, the authors found that sourdough retards staling, protects the bread from spoilage, and contributes to an extended shelf life [45]. Sourdough LAB shows antibacterial, antimicrobial, and antifungal activity by releasing different metabolites that can substitute chemical preservatives. Lactic acid bacteria (LAB) release antifungal metabolites with low-molecular-mass compounds such as cyclic dipeptides, hydroxyl fatty acids, phenyls, and substituted phenyl derivatives. Another antifungal mechanism of LAB is producing mixtures of organic acids such as acetic, butyric, caproic, formic, *n*-valeric, and propionic acids [45].

#### 5.2.2. Essential Oils

Plants can combat pathogen infections by different compounds that can be separated into three categories: phytoanticipins, which are antimicrobial components; inducible performed compounds; and phytoalexins, which are inhibitory components synthesized when the plant feels attacked. All these compounds have a bio-preservative activity useful in the food industry. However, the one with more research are the antimicrobial components, where essential oils are the most significant group. There are around 3000 essential oils, but only 300 have commercial importance in the food, pharmaceutical, agronomic, and cosmetic industries. Essential oils’ antimicrobial activity is influenced by their composition, concentration, structure, and functional groups [42,46]. In addition, there are four groups of active compounds: terpenes, terpenoids, phenylpropenes, and others. There are different tests to evaluate the antifungal activity of essential oils. Some of the assays are done in vitro and performed in three different ways: diffusion assay, dilution assay, and poisoned food assay. The most common is diffusion assay in different media. It is used to express antifungal activity by the zone of inhibition of the fungi surrounding the filter paper or the active component. Most of these in vitro assays can sometimes underestimate essential oils’ antifungal activity by just looking for the minimum inhibitory concentration (MIC) [44].

In bread making, researchers used different essential oils in the dough or after baking. However, most of the potential fungal activity is lost during the heat treatment in baking, oxidation, and decrease in bioavailability [44]. The use of rosemary essential oils inhibits the growth of *Penicillium* sp. and *Aspergillus* sp. at an oil concentration of 50 µm/mL in fresh dough [46]. This study used a control (no oil), different oil concentrations, and microencapsulated oil treatments to compare its microbial inhibition. The microencapsulated essential oil showed that regardless of heat, it does not interfere with the oil constituents. It showed a better result in prolonged inhibition when added at 1.5% to fresh dough. Microencapsulation showed better inhibition over time, maybe due to a gradual release of the active components, with a lower fungal count after 8 and 12 days of storage compared to pure oil [46].

In other research, clove bud (*Syzygium aromaticum*) and oregano (*Origanum vulgare*) essential oils have been used as antimicrobial preservatives to extend the shelf life of sliced bread. In this study, the authors used different sizes of emulsions of the essential oils in a methylcellulose film and then counted the yeasts and molds in sliced bread during the 15 days of storage at 25 ± 2 °C. Table 2 shows the results of the inhibition halo of *Aspergillium niger* and *Penicillium* sp., with different concentrations of the essential oils for the film [47].

#### 5.2.3. Packaging Preservation

**Figure 3:**
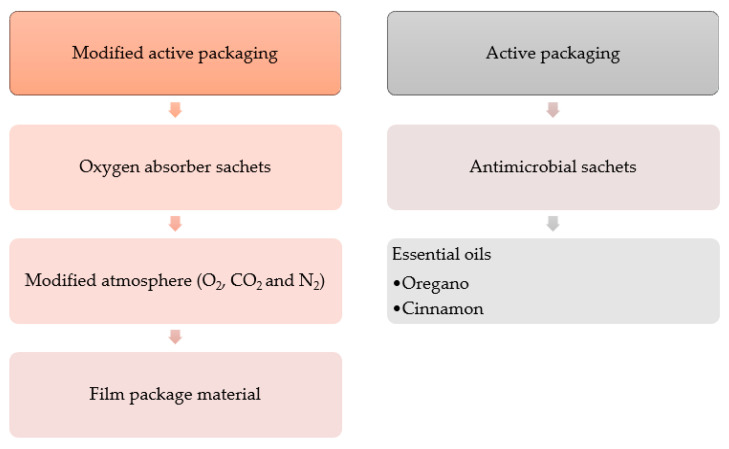
Summary of packaging materials to extend bread’s shelf life.

Intending to have a clean-label bread formulation are also studies changing the packaging type and using other types of additives in formulations (Figure 3). The packaging industries came out with strategies to extend the shelf life with modified atmosphere packaging (MAP) and active packaging [44]. Modified atmosphere packaging is defined as the displacement of gases inside the package and their replacement by the desired mixture of gases or a natural result of the selected film type and product respiration [49]. Active packaging consists of applying active agents directly to the packaging material instead of the food, enhancing food quality and safety [9].

In the packaging industry, different materials are used to help extend bread’s shelf life. Film packages with an oxygen barrier and a modified packaging atmosphere can prolong the shelf life from 3–4 days to 5–7 days [48]. In addition, in this study, to achieve this modified atmosphere, oxygen absorber sachets lasting for 4 days were used. The modified atmosphere reduced fungi and mold development to a greater degree than the treatments without a modified atmosphere. The three-layer, low-density polyethylene (LDPE) films with O-nylon lamination had a better result in terms of the film package but with a low O_2_ atmosphere of 5% (*v*/*v*) [48]. Other studies have shown that a modified packaging atmosphere of 100% N_2_ and 100% CO_2_ also effectively extends the shelf life to 13–24 days, but with a 60% CO_2_ atmosphere, there is a change to a more acidic taste in the bread [50].

Other researchers have had the idea of using antimicrobial essential oil (EO) sachets in packages containing sliced bread [9,43]. Oregano essential oil has proved to have antimicrobial properties in controlling pathogenic microorganisms such as *Penicillium* sp., *Aspergillus* sp., *Staphylococcus* sp., *Bacillus* sp., *E.* *coli*, and *Salmonella enteriditis* [51,52]. One of the major components of oregano essential oil with antimicrobial activity is carvacrol, which has better performance in vapor form. The sachets slowly release the essential oil properties. A metalized polypropylene bag, different from the commercially available bag that is more permeable to gases, was used in [9]. The oregano essential oil sachets were successful in controlling *Penicillium* sp. and other fungal spoilage. As the concentration increased, the microbial growth rate decreased, but all concentrations had the same count of yeasts and molds at the end of the 15 days in the sliced bread [9]. There was no change in the bread texture after the use of oregano essential oil, but there were unpleasant sensory effects at the highest oregano oil concentration [9]. In addition, sachets with cinnamon EO combined with micro-perforated polypropylene packaging material could increase the shelf life of bread from 3 to 10 days, according to Gutierrez et al. [43]. In this research, the authors used known concentrations of cinnamon EO in propylene films. Other studies have shown that cinnamon EO at 6% can completely inhibit the growth of microorganisms such as *Rhizopusstolonifer stolonifera*, where the compound cinnamaldehyde is mostly found [53].

## 6. Conclusions and Future Challenges

The clean-label trend has become challenging for the baking industry and small bakeries. The food industry must respond to consumers’ demands for more natural ingredients without losing the bread’s taste, texture, and quality. Although there is no formal and standardized definition of a clean label, consumers still agree that the product should be healthy and have no chemical-sounding names in the ingredient list. Other research has focused on using different dough strengtheners and has shown that it is feasible to use other flour and wheat gluten. Dough properties such as water absorption, extensibility, strength, softer crumb, and loaf volume can be enhanced. Enzymes have shown a possible replacement for dough strengtheners such as hydrocolloids and emulsifiers. In the case of preservatives, there are investigations on fermenters and essential oils but with some challenges. As an option for extending bread shelf life, solutions as active packaging and modified active packaging are evaluated. The use of cinnamon and oregano essential oil has shown inhibition in microbial growth in bread.

In future research, it is essential not only to focus on the dough rheology but also to conduct a sensory evaluation. The use of essential oils has shown an effect on the final product’s taste, which can lower consumer acceptance. Another part that needs evaluation is whether these technologies as substitutes for dough strengtheners and preservatives are feasible in price and manufacturing matters. Some of the options for extending bread shelf life and as strengtheners are considered expensive or cannot be used on a larger scale in bakeries.

In conclusion, after proving an effective clean-label formulation, it is vital to assess the final product’s taste and texture and evaluate its replication in small- and large-scale bakeries. The future challenge is to keep investigating feasible and practical replacements of dough strengtheners and preservatives that bakers can use.

## Figures and Tables

**Table 1:** Dough-conditioning agents with functions and use levels [16].

Dough Conditioner Ingredient	Function	Usage Level	Considerations
Vital wheat gluten	Structure	2% to 10%	Increases strength and absorption
Ammonium chloride	Yeast nutrient	0.04%	Nitrogen source
Ammonium sulfate	Yeast nutrient	0.04%	Nitrogen source
Ammonium phosphate	Yeast nutrient	0.04%	Nitrogen and phosphorus source
Calcium carbonate	pH regulator	0.1% to 0.5%	Raises pH
Monocalcium phosphate	pH regulator	0.1% to 0.3%	Lowers pH
Calcium sulfate	pH regulator	0.1% to 0.6%	Raises pH
Potassium bromate	Oxidizing agent	10 to 75 ppm	Slow oxidizer
Ascorbic acid	Oxidizing agent	10 to 100 ppm	Intermediate oxidizer
Calcium peroxide	Oxidizing agent	10 to 75 ppm	Dries dough surface
Azodicarbonamide	Oxidizing agent	10 to 45 ppm	Fast oxidizer
Potassium iodate	Oxidizing agent	10 to 75 ppm	Fast oxidizer
Calcium iodate	Oxidizing agent	10 to 75 ppm	Fast oxidizer
L-cysteine	Reducing agent	10 to 90 ppm	Chemical reducing agent
Non-leavening (inactive) yeast	Reducing agent	0.25% to 1%	Natural source of glutathione
Protease	Enzyme	GMP ^1^	Increases extensibility
Carbohydrase	Enzyme	GMP	Improves oven spring and freshness
Oxidase	Enzyme	GMP	Forms oxygen via hydrogen peroxide
Enzyme-active soy flour	Enzyme	0.25% to 0.5%	Lipoxygenase whitens crumb
Diastatic malt syrup	Enzyme	1% to 2%	Supplements flour enzyme activity
Malt flour	Enzyme	0.5% to 1%	Supplements flour enzyme activity
Lecithin	Emulsifier	0.25% to 1%	Natural softener
Sodium stearoyl lactylate	Emulsifier	0.25% to 0.5%	Strengthens and softens
Calcium stearoyl lactylate	Emulsifier	0.25% to 0.5%	Strengthens and softens
Diacetyl tartaric acid esters of mono- and diglycerides (DATEM)	Emulsifier	0.25% to 0.5%	Strengthens
Ethoxylated mono- and diglycerides	Emulsifier	0.25% to 0.5%	Strengthens
Polysorbate 60	Emulsifier	0.25% to 0.5%	Softens
Succinylated mono- and diglycerides	Emulsifier	0.25% to 0.5%	Strengthens and softens
Mono- and diglycerides	Emulsifier	0.25% to 1%	Softens
Distilled diglycerides	Emulsifier	0.25% to 1%	Softens

^1^ Good manufacturing practice.

**Table 2:** Inhibition halos of *Aspergillus niger* or *Penicillium* sp. after 5 days of storage at 25 ± 2 °C, as affected by clove bud or oregano essential oils [48].

	Clove Bud Essential Oil		Oregano Essential Oil	
Concentration (mg mL^−1^)	*Aspergillus niger*	*Penicillium* sp.	*Aspergillus niger*	*Penicillium* sp.
40.0	34.43 ± 2.10 a ^1^	30.71 ± 3.50 a	29.24 ± 1.20 a	27.59 ± 2.05 a
20.0	15.93 ± 10.20 b	13.10 ± 12.25 a	15.85 ± 12.44 b	9.72 ± 10.77 b
10.0	0.00 ± 0.00 c	0.00 ± 0.00 b	0.00 ± 0.00 c	0.00 ± 0.00 c
5.00	0.00 ± 0.00 c	0.00 ± 0.00 b	0.00 ± 0.00 c	0.00 ± 0.00 c
2.50	0.00 ± 0.00 c	0.00 ± 0.00 b	0.00 ± 0.00 c	0.00 ± 0.00 c
1.25	0.00 ± 0.00 c	0.00 ± 0.00 b	0.00 ± 0.00 c	0.00 ± 0.00 c
0.00	0.00 ± 0.00 c	0.00 ± 0.00 b	0.00 ± 0.00 c	0.00 ± 0.00 c

^1^ Values in the same column with the same letter are not significantly different, *p* < 0.05.

## Data Availability

This study does not report any data.
